# Cancer stem cell assay-guided chemotherapy improves survival of patients with recurrent glioblastoma in a randomized trial

**DOI:** 10.1016/j.xcrm.2023.101025

**Published:** 2023-05-02

**Authors:** Tulika Ranjan, Soma Sengupta, Michael J. Glantz, Richard M. Green, Alexander Yu, Dawit Aregawi, Rekha Chaudhary, Ricky Chen, Mario Zuccarello, Christine Lu-Emerson, Hugh D. Moulding, Neil Belman, Jon Glass, Aaron Mammoser, Mark Anderson, Jagan Valluri, Nicholas Marko, Jason Schroeder, Steven Jubelirer, Frances Chow, Pier Paolo Claudio, Anthony M. Alberico, Seth T. Lirette, Krista L. Denning, Candace M. Howard

**Affiliations:** 1 Department of Neuro-Oncology, Allegheny Health Network, Pittsburgh, PA, USA; 2 Department of Neuro-Oncology, Cancer Center Southern Florida, Tampa General Hospital, Tampa, FL, USA; 3 Department of Neurology and Rehabilitation Medicine, University of Cincinnati, Cincinnati, OH, USA; 4 Department of Neurosurgery, Penn State Neuroscience Institute, Hershey, PA, USA; 5 Department of Neuro-Oncology, Southern California Permanente Medical Group, Los Angeles, CA, USA; 6 Department of Neurosurgery, Allegheny Health Network, Pittsburgh, PA, USA; 7 Department of Internal Medicine, Division of Hematology-Oncology, University of Cincinnati, Cincinnati, OH, USA; 8 Department of Neuro-Oncology, Providence Brain & Spine Institute, Portland, OR, USA; 9 Department of Neurosurgery, University of Cincinnati, Cincinnati, OH, USA; 10 Department of Neuro-Oncology, Maine Medical Center, Scarborough, ME, USA; 11 Department of Neuroscience, St. Luke’s University Hospital & Health Network, Bethlehem, PA, USA; 12 Departments of Neurology and Neurological Surgery, Thomas Jefferson University, Philadelphia, PA, USA; 13 Department of Neurosurgery, LSU Health Sciences Center, New Orleans, LA, USA; 14 Department of Neurology, University of Mississippi Medical Center, Jackson, MS, USA; 15 Cordgenics, LLC, Huntington WV, USA; 16 Department of Pharmacology & Toxicology, University of Mississippi Medical Center, Jackson, MS, USA; 17 Department of Neurosurgery, LewisGale Regional Health System, Salem, VA, USA; 18 Department of Neurosurgery, University of Toledo, Toledo, OH, USA; 19 Department of Neuro-Oncology, Charleston Area Medical Center, Charleston, WV, USA; 20 Departments of Neurological Surgery and Neurology, University of Southern California, Los Angeles, CA, USA; 21 Department of Neurosurgery, Joan C. Edwards School of Medicine, Marshall University, Huntington, WV, USA; 22 Department of Data Science, University of Mississippi Medical Center, Jackson, MS, USA; 23 Department of Pathology, Joan C. Edwards School of Medicine, Marshall University, Huntington, WV, USA; 24 Department of Radiology, University of Mississippi Medical Center, Jackson, MS, USA

**Keywords:** glioblastoma, recurrent glioblastoma, ChemoID, personalized medicine, cancer stem cells, assay-guided chemotherapy, functional assay, drug response assay, chemotherapy, overall survival

## Abstract

Therapy-resistant cancer stem cells (CSCs) contribute to the poor clinical outcomes of patients with recurrent glioblastoma (rGBM) who fail standard of care (SOC) therapy. ChemoID is a clinically validated assay for identifying CSC-targeted cytotoxic therapies in solid tumors.

In a randomized clinical trial (NCT03632135), the ChemoID assay, a personalized approach for selecting the most effective treatment from FDA-approved chemotherapies, improves the survival of patients with rGBM (2016 WHO classification) over physician-chosen chemotherapy. In the ChemoID assay-guided group, median survival is 12.5 months (95% confidence interval [CI], 10.2–14.7) compared with 9 months (95% CI, 4.2–13.8) in the physician-choice group (p = 0.010) as per interim efficacy analysis. The ChemoID assay-guided group has a significantly lower risk of death (hazard ratio [HR] = 0.44; 95% CI, 0.24–0.81; p = 0.008). Results of this study offer a promising way to provide more affordable treatment for patients with rGBM in lower socioeconomic groups in the US and around the world.

## Highlights

• Cancer stem cell assay (ChemoID) can accurately predict clinical response to treatment
• ChemoID assay is evaluated in a randomized clinical trial for recurrent glioblastoma
• FDA-approved chemotherapies for recurrent glioblastoma are utilized in the trial
• ChemoID assay-guided therapy improves the median OS and PFS of patients with recurrent GBM

## Graphical abstract

**Figure:**
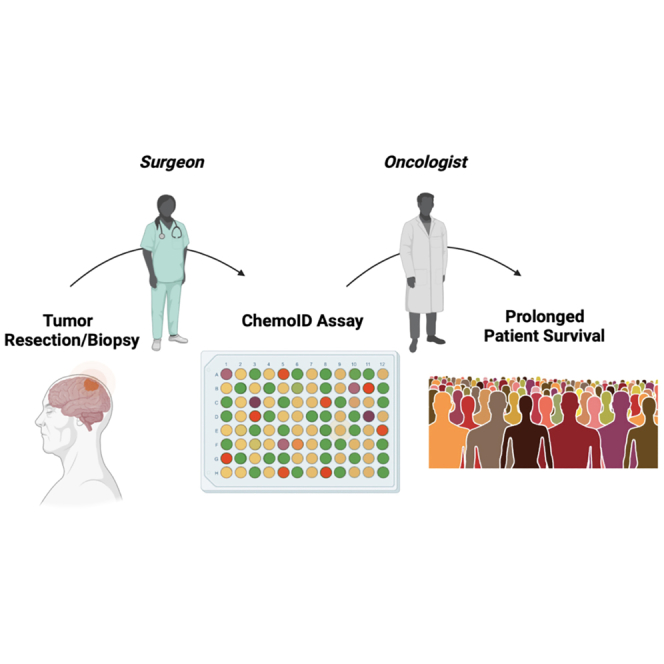

## Teaser

Ranjan et al. use a cancer stem cell assay to guide treatment for recurrent GBM. Targeting cancer stem cells and bulk of tumor cells from patient biopsies to predict the clinical response improves survival outcomes. Results of this clinical trial represent the value of functional diagnostics in precision oncology.

## Introduction

The standard of care (SOC) for glioblastoma (GBM) is a combination of surgery, radiotherapy, and concomitant temozolomide followed by maintenance temozolomide (TMZ) as demonstrated by the EORTC-NCIC trial.^1^^,^^2^ Treatment of recurrent GBM (rGBM) is most commonly single or combination chemotherapy with nitrosoureas, TMZ, CPT-11, or bevacizumab (BV).^1^^,^^2^^,^^3^ Unfortunately, current treatment options have proven to be largely ineffective, with some drugs having little to no survival benefit.^4^^,^^5^^,^^6^^,^^7^^,^^8^ Patients have a poor prognosis as GBM generally recurs with a 5-year survival rate of less than 10%.^1^^,^^2^^,^^7^^,^^8^^,^^9^ There is a significant unmet need for new strategies to personalize therapeutic options for the treatment of rGBM.

Due to the unpredictable nature of cancer, responses to chemotherapy can vary from patient to patient, even when cancer cells are of the same histology. Several genome-based methodologies and immunotherapies are under clinical investigation. They have not demonstrated a survival advantage in GBM,^10^ and genome-directed drugs are often only applicable to a subset of patients with cancer with unique biomarkers.^11^^,^^12^ Many patients with cancer in the US in lower socioeconomic groups struggle to pay for their treatments because of rising costs for novel treatments and rising premiums, deductibles, and copayments for private health insurance plans.^13^ Additionally, most people, particularly in underserved areas, including those in developed nations with socialized healthcare systems or underdeveloped nations, lack access to many of the more recent targeted therapies and immunotherapies.^14^ Also, immunotherapies, as yet, have not shown a survival advantage in GBM.^10^ For these reasons, developing and optimizing cost-effective cytotoxic chemotherapies remain important.

A strategy to increase the survival of patients with GBM is to target the cancer stem cells (CSCs) that contribute to therapy resistance and cancer progression^11^^,^^12^ by utilizing first- and second-line cytotoxic chemotherapies routinely covered by Medicare and health insurance plans. While there are newer targeted therapies and immunotherapies available today, this trial focused on screening SOC chemotherapies that are routinely covered and available to community oncology patients and for patients from other countries, where the more novel agents are not readily available.

The “CSC concept” was proposed four decades ago and revised recently and posits that tumor growth is analogous to the renewal of healthy tissues and fueled by small numbers of dedicated stem cells capable of plasticity.^11^^,^^15^^,^^16^^,^^17^ The unidirectional and irreversible hierarchical progression of GBM growth has been studied in an animal model of GBM.^18^ The ablation of CSCs in this model halted tumor growth and prolonged survival without apparent regeneration of the CSC pool from other GBM cells.^18^ Many of the current therapeutic strategies (chemotherapy and radiation) aimed at eliminating rapidly dividing cancer cells (bulk tumor) involve treatment with standard anti-proliferative chemotherapy, with limited response. This poses a challenge, as the residual population of chemotherapy-resistant tumor cells capable of regenerating the disease (relapse) is enriched in CSCs.^11^^,^^19^ In recent years, genetic fate mapping in several types of solid tumors has supported the notion that recurrence after chemotherapy results from the persistence of CSCs. Slow-proliferating CSCs of GBM resist TMZ treatment in animal models. Genetic ablation of this cell population renders GBMs susceptible again to chemotherapy.^18^ These data suggest that differentiated cells may subsequently replace lost stem cells through plasticity inferring that the CSCs, by representing a source of chemotherapy-resistant cells, contribute to the occurrence of relapse after treatment.^15^^,^^19^

For patients with rGBM, a viable treatment option is to select chemotherapies that will eliminate the CSCs, the main cause of treatment resistance, while reducing the bulk of tumor cells.^19^ Real-world clinical studies demonstrated improved survival of patients with rGBM after treatment with CSC assay-guided chemotherapy regimens (ChemoID).^20^^,^^21^

Based on this proof of concept and on real-world data, a multi-institutional, randomized clinical trial (ClinicalTrials.gov: NCT03632135) of patients with rGBM was initiated to assess the efficacy of chemotherapy regimens selected by the ChemoID assay vs. best physician choice. The primary efficacy endpoint of this trial was demonstrated at a prespecified interim efficacy analysis.

## Results

**Figure 1:**
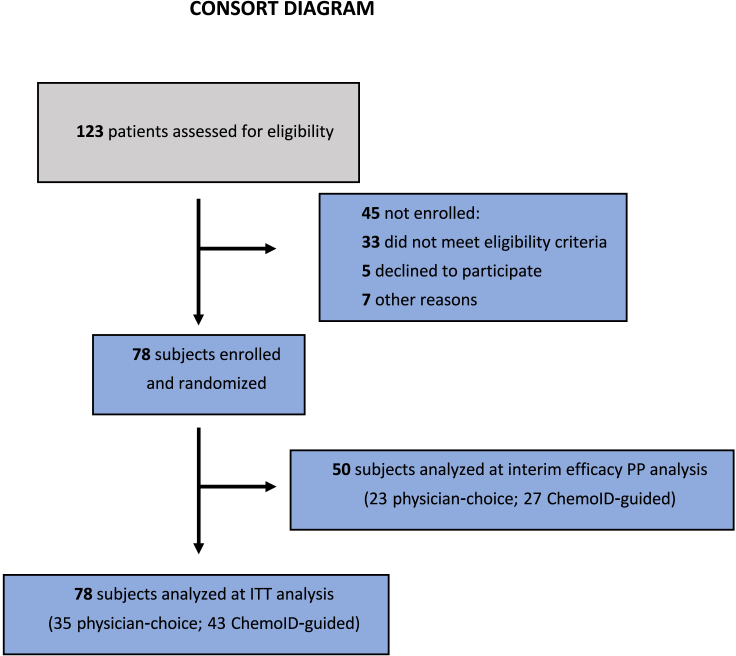
CONSORT diagram of ChemoID study A total of 123 patients were screened between May 18, 2018, and May 30, 2021; 78 of these patients were randomized to either the ChemoID or physician-choice group. After 35 deaths were reported, the first prespecified interim efficacy analysis was performed.

**Figure 2:**
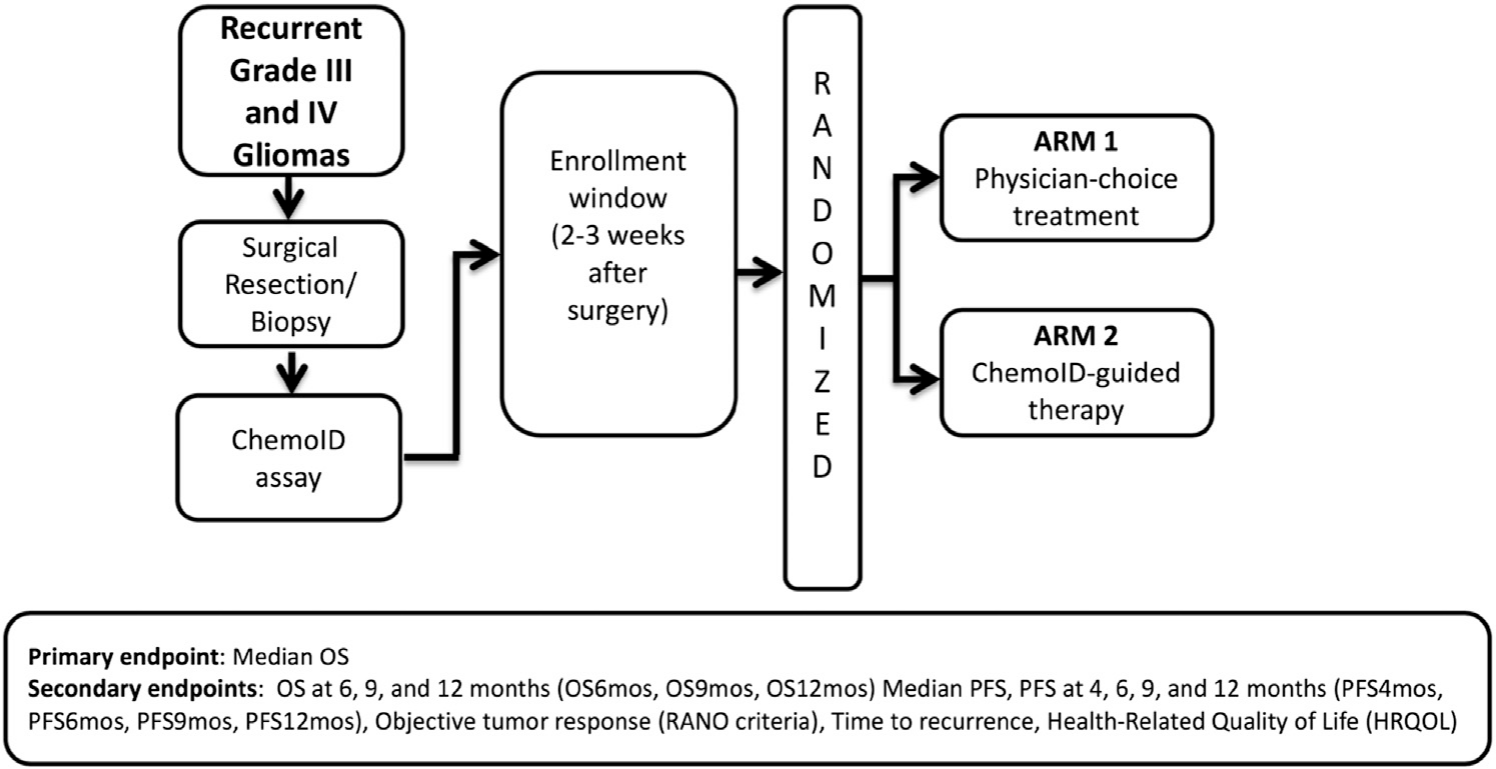
Study schema of the registered clinical trial NCT03632135 A multi-institutional, randomized clinical trial of patients with rGBM was initiated to assess the efficacy of chemotherapy regimens selected by the ChemoID assay vs. best physician choice. The primary efficacy endpoint of this trial was median OS. Secondary endpoints were OS at 6, 9, and 12 months, median PFS at 4, 6, 9, and 12 months, objective tumor response, time to recurrence, and health-related quality of life.

Over a period of 3 years, 123 patients affected by rGBM or grade III glioma were screened and assessed for eligibility criteria to participate in a parallel-group-randomized controlled trial at 13 clinical sites across the US. The study protocol was approved by the Western Institutional Review Board (WIRB) and the independent ethics committee of each of the participating institutions. 78 patients with rGBM (diagnosed according to the 2016 WHO classification of brain tumors) were enrolled in the study (consort diagram: Figure 1) after signing the informed consent. All enrolled subjects underwent surgical resection and biopsy. For histopathology confirmation and diagnosis, MGMT gene methylation, and IDH-1/-2 status, the tumor biopsies were sent to the sites’ hospital pathology laboratory. A portion of each of the biopsies was sent from the operating room as a fresh tumor sample to the ChemoID central clinical pathology laboratory, where the assay was conducted. The ChemoID assay is a diagnostic test that determines the cytotoxic profile of CSCs and the bulk of tumor cells treated with various NCCN-approved chemotherapies and/or their combination. All registered patients in the trial underwent the ChemoID assay and were randomly assigned by the sites’ coordinators to a study group using a computer-generated algorithm (in REDCap). Subjects were treated either with SOC chemotherapy chosen by the physician or treatment directed by the ChemoID assay (Figure 2). The treatment regimens in the two groups were chosen from the same list of chemotherapies (Table S1), depending on the randomly allocated study group (assay guided or physician best choice).

A predetermined interim survival efficacy analysis was conducted when 35 deaths were reported (August 20, 2021). Patient enrollment was stopped after the interim efficacy analysis because the study’s primary endpoint (overall survival [OS]) was met during the planned interim efficacy analysis. Intention-to-treat (ITT) analysis was conducted on all 78 randomized subjects.

The median time of follow-up was 10.5 months for the ChemoID assay-guided group and 6.2 months for the physician-choice group at the time of the interim efficacy analysis data cutoff. The median time of follow-up at ITT analysis was 10.5 months for the ChemoID assay-guided group and 7.5 months for the physician-choice group.

A post-randomization analysis of the demographics and baseline clinical characteristics of all randomized subjects demonstrated that subjects were balanced between the two study groups (Table 1). *MGMT* methylation status and *IDH1*/*IDH2* gene status (mutated vs. wild type) indicated that in both arms, most patients had unfavorable prognoses (i.e., unmethylated *MGMT* promoter and wild-type *IDH1*/*IDH2*). None of the patients used the NovoTTF-100L system (Optune) during the study.

**Table 1:** Patient demographics and baseline characteristics

	Physician choice (n = 35)	ChemoID guided (n = 43)	p value
Age, mean (SD)	57.5 (10.7)	57.9 (13.1)	0.887
Male, no. (%)	21 (60)	28 (65)	0.814
Non-White, no. (%)	4 (11)	7 (16)	0.746

Histopathologic diagnosis,^a^ no. (%)			

Recurrent glioblastoma	35 (100)	43 (100)	>0.999

MGMT promoter methylation status, no. (%)			

Methylated	11 (31.4)	15 (34.8)	0.812
Unmethylated	24 (68.6)	28 (65.2)	0.812

IDH1/IDH2 status, no. (%)			

Mutant	3 (8.5)	7 (16.2)	0.498
Wild type	32 (91.4)	36 (83.7)	0.498
Measurable lesions, no.	50	66	NA
Target lesion size, median (range), mm^2^	500 (200–1700)	500 (200–1900)	>0.999

Site of target lesion(s), no. (%)			

Temporal lobe	22 (44)	27 (41)	0.731
Frontal lobe	8 (16)	15 (23)	0.731
Parietal lobe	12 (24)	12 (18)	0.731
Occipital lobe	3 (6)	7 (11)	0.731
Parietal and occipital	5 (10)	5 (8)	0.731
Cerebellum	0	0	0.731
Brain stem	0	0	0.731
Insula	0	0	0.731

Karnofsky performance status at study entry, no. (%)			

≤80	0	0	>0.999
≥80	35 (100)	43 (100)	>0.999

Corticosteroid use, no. (%)			

≤2 mg/day	2 (5.7)	4 (9.3)	0.879
>2 to <4 mg/day	9 (25.7)	9 (21)	0.879
≥4 mg/day	6 (17.1)	6 (13.9)	0.879
No	18 (51.5)	24 (55.8)	0.879

a Glioblastomas were diagnosed following the 2016 WHO classification.^12^

### ChemoID assay-guided therapy increased OS and progression-free survival (PFS) of patients with rGBM

**Figure 3:**
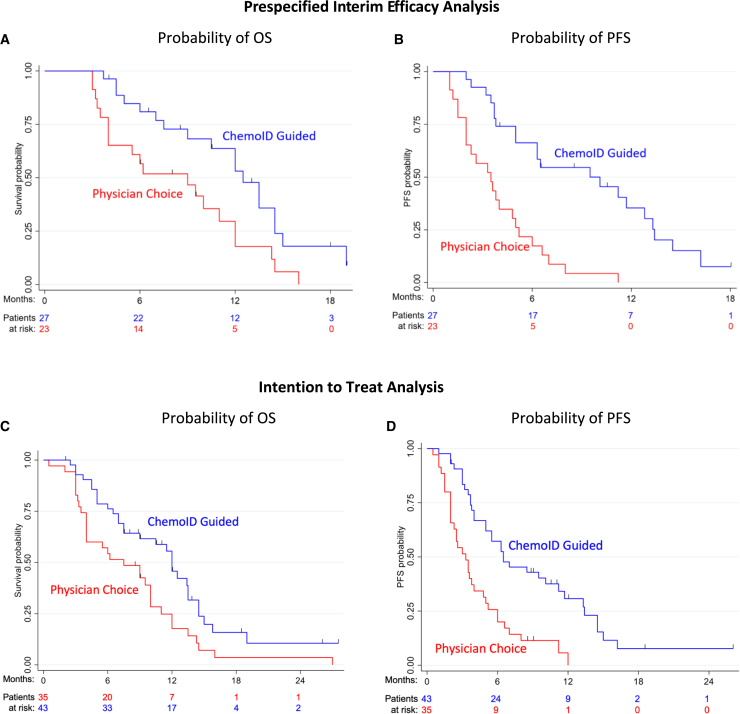
OS and PFS are significantly improved by ChemoID-guided therapy (A) Prespecified interim efficacy analysis of OS. The number of events; median OS; OS rates at 0, 6, 12, and 18 months; and the Kaplan-Meier curve for OS in all patients treated with ChemoID-guided (blue) vs. physician-choice (red) therapies. Symbols, censored observations. (B) Prespecified interim efficacy analysis of PFS. The number of events; median PFS; PFS rates at 0, 6, 12, and 18 months; and the Kaplan-Meier curve for PFS per investigator assessment in patients treated with ChemoID-guided (blue) vs. physician-choice (red) therapies. Symbols indicate censored observations. (C) Intention-to-treat analysis of OS. The number of events; median OS; OS rates at 0, 6, 12, and 18 months; and the Kaplan-Meier curve for OS in all patients treated with ChemoID-guided (blue) vs. physician-choice (red) therapies. Symbols, censored observations. (D) Intention-to-treat Analysis of PFS. The number of events; median PFS; PFS rates at 0, 6, 12, and 18 months; and the Kaplan-Meier curve for PFS per investigator assessment in patients treated with ChemoID-guided (blue) vs. physician-choice (red) therapies. Symbols indicate censored observations. A Cox proportional hazards model estimated hazard ratios (HRs) and CIs.

A statistically significant difference was observed in the risk of death between groups (hazard ratio [HR] = 0.44; 95% confidence interval [CI], 0.24–0.81; p = 0.008) in the interim efficacy analysis. In the ChemoID assay-guided group, 67% of patients (18 of 27) died vs. 87% (20 of 23) in the physician-choice group. Furthermore, median overall survival (mOS) was 12.5 months (95% CI, 10.2–14.7) with ChemoID assay-guided therapy vs. 9 (95% CI, 4.2–13.8) with physician choice (log rank p = 0.010) (Figure 3A). As a secondary endpoint, survival was analyzed at 6, 9, and 12 months. The probability of survival for the ChemoID assay group was 0.85 vs. 0.61 for the physician-choice group at 6 months (odds ratio [OR], 3.53; 95% CI, 0.91–13.7; p = 0.068), 0.70 vs. 0.48 at 9 months (OR, 2.51; 95% CI, 0.73–8.63; p = 0.143), and 0.57 vs. 0.25 at 12 months (OR, 4; 95% CI, 1.06–15.1; p = 0.041) (Table 2).

**Table 2:** Secondary endpoints: Interim efficacy analysis of OS and PFS probability

OS (months)	OS probability, ChemoID guided	OS probability, physician choice	Odds ratio (OR)	95% CI	p value
6	0.85	0.61	3.53	0.91–13.7	0.068
9	0.70	0.48	2.51	0.73–8.63	0.143
12	0.57	0.25	4.00	1.06–15.1	0.041

PFS (months)	PFS probability, ChemoID guided	PFS probability, physician-choice	OR	95% CI	p value
6	0.65	0.22	6.80	1.89–24.4	0.003
9	0.50	0.04	22.00	2.54–190.0	0.005
12	0.30	0.00	NC^a^	NC^a^	NC^a^

a NC, not calculable.

The median progression-free survival (mPFS) was 10.1 months (95% CI, 4.8–15.4) for patients receiving ChemoID assay-guided therapy vs. 3.5 months (95% CI, 1.9–5.1) for physician-choice therapy (HR, 0.25; 95% CI, 0.14–0.44; p < 0.001) (Figure 3B). The probability of PFS was greater in the ChemoID assay-guided group vs. the physician-choice group: 0.65 vs. 0.22 at 6 months (OR, 6.80; 95% CI, 1.89–24.4; p = 0.003); 0.50 vs. 0.04 at 9 months (OR, 22.0; 95% CI, 2.54–190; p = 0.005); and 0.3 vs. 0 at 12 months (all patients in the physician-choice group had progressed) (Table 2).

ChemoID assay-guided therapy continued to demonstrate meaningful clinical benefit in mOS throughout follow-up. In ITT analysis, a statistically significant difference was also observed in the risk of death between groups (HR = 0.52; 95% CI, 0.24–0.81; p = 0.008). In the ChemoID assay-guided group, 70% of patients (30 of 43) died vs. 91% (32 of 35) in the physician-choice group. Furthermore, mOS was 12 months (95% CI, 10.8–13.2) with ChemoID assay-guided therapy vs. 7.5 (95% CI, 3.5–11.5) with physician choice (log rank p = 0.009) (Figure 3C). Survival was analyzed at 6, 9, and 12 months as a secondary endpoint. The probability of survival for the ChemoID assay group was 0.79 vs. 0.57 for the physician-choice group at 6 months (OR, 2.75; 95% CI, 1.02–7.44; p = 0.046), 0.62 vs. 0.47 at 9 months (OR, 1.80; 95% CI, 0.71–4.57; p = 0.217), and 0.49 vs. 0.22 at 12 months (OR, 3.37; 95% CI, 1.16–9.82; p = 0.026) (Table 3).

**Table 3:** Secondary endpoints: ITT analysis of OS and PFS probability

OS (months)	OS probability, ChemoID guided	OS probability, physician choice	Odds ratio (OR)	95% CI	p value
6	0.79	0.57	2.75	1.02–7.44	0.046
9	0.62	0.47	1.80	0.71–4.57	0.217
12	0.49	0.22	3.37	1.16–9.82	0.026

PFS (months)	PFS probability, ChemoID guided	PFS probability, physician choice	OR	95% CI	p value
6	0.57	0.26	3.85	1.45–10.2	0.007
9	0.42	0.09	7.32	1.92–27.9	0.004
12	0.24	0.03	10.3	1.23–86.3	0.032

The mPFS was 6.5 months (95% CI, 3.3–9.7) for patients receiving ChemoID assay-guided therapy vs. 3.3 months (95% CI, 2.1–4.5) for physician-choice therapy (HR, 0.36; 95% CI, 0.23–0.57; p < 0.001) (Figure 3D). The probability of PFS was greater in the ChemoID assay-guided group vs. the physician-choice group: 0.57 vs. 0.26 at 6 months (OR, 3.85; 95% CI, 1.45–10.2; p = 0.007), 0.42 vs. 0.09 at 9 months (OR, 7.32; 95% CI, 1.92–27.9; p = 0.004), and 0.24 vs. 0.03 at 12 months (OR, 10.3; 95% CI, 1.23–86.3; p = 0.032) (Table 3).

### Exploratory analyses

#### ChemoID test results correlated with patients’ OS and PFS

Survival and PFS of each patient in the planned interim efficacy analysis were also analyzed as a function of the cell kill of the patient’s cultured tumor cells (both CSCs and bulk tumor cells) in response to the drug(s) used during treatment (Figure S1). Logistic regression models were constructed based on the ChemoID assay report data of patients’ cultured CSCs and bulk tumor cells exposed to the same drug(s) used during their treatment. We found that the optimal thresholds of tumor cell kill were 40% for CSCs and 55% for bulk tumor cells as per the logistic regression models (see referent lines in Figure S1). These thresholds agree with our previously published data.^20^^,^^21^^,^^22^ For patients in the physician-choice arm, data points were broadly distributed over both axes, as expected given that treating physicians were blinded to the ChemoID data for patients in this arm. In striking contrast, most data points for patients in the ChemoID arm were clustered in the upper right quadrant (i.e., high percentage kill of both CSCs and bulk tumor cells).

Our analysis further revealed that for every 10% increase in CSC drug response, there was a significant increase (13%) in 6-month patient survival (HR, 0.87; p = 0.012), and for every 10% increase in bulk tumor cell response, the hazard of death decreased 13% (HR, 0.87; p = 0.024). We also found that for every 10% increase in cell kill, the hazard of progression at 6 months decreased by 14% for CSCs (HR, 0.86; p = 0.005) and 18% for bulk tumor cells (HR, 0.82; p = 0.001).

For survival trials, HR is the standard reporting mechanism; however, restricted mean survival time (RMST) is also a robust method for assessing the treatment effect.^23^ We have analyzed the data using the 1- or 1.5-year RMST and CIs for each of our primary results to quantify the treatment effect and found that for the interim analysis, the mOS was 3.36 months (1.03–5.69) and the mPFS was 4.87 months (2.64–7.10). Instead for the ITT analysis, the mOS was 2.93 months (0.76–5.11), and the mPFS was 4.37 months (2.36–6.39). Additionally, we performed several Cox-Snell residual proportionality tests with the collected data, and all p values were 0.38 or greater.

In our exploratory studies, we also analyzed the cohort of patients with rGBM by removing subjects affected by *IDH1*/*IDH2* mutations. We found there was no effect on OS or PFS outcomes when subjects affected by IDH1/IDH2 mutations were removed from the analysis.

The trial allowed the optional inclusion of BV during treatment because it has been shown in large trials and meta-analyses that BV treatment in combination with chemotherapy improves the management of symptoms and quality of life in patients with rGBM but not the OS.^4^^,^^24^ We conducted an exploratory analysis stratifying in each arm the patients who had BV treatment along with chemotherapy and found that the use of BV in the ChemoID-guided group did not contribute to an advantage in their survival (11.5 months with BV vs. 12.5 months without BV). The survival in the ChemoID group was greater when subjects did not receive BV compared with subjects in the physician-choice group who did receive BV (12.5 months without BV vs. 10 months with BV), indicating that the OS advantage observed in the ChemoID-guided group was due to the use of the most effective chemotherapy regimen, independent from the use of BV (Figure S2A). Similarly, the use of BV in the ChemoID-guided group did not contribute to an advantage in their PFS (6.3 months with BV vs. 7 months without BV) (Figure S2B).

### Correlation between chemotherapy treatments administered and the ChemoID test report predictions

The drug response to each chemotherapy and their combinations were analyzed to determine the proportion of patients who benefitted from a sensitive vs. non-sensitive chemotherapy chosen prospectively by the ChemoID assay. A pyramid diagram representation of the comparison in percent of cell kill of the most cytotoxic drugs found by the ChemoID assay compared with the actual cell kill percentages of the chemotherapy treatment used for both physician-choice and ChemoID-guided groups for each patient is shown in Figure S3. Optimal therapies with the highest cell kill found by the ChemoID assay are shown in light colors and actual therapies used are shown in dark colors, with each row of the pyramid corresponding to results for a single patient. Results from the physician-choice group are shown in red, while those from the ChemoID-guided group are shown in blue. In Figure S3, the pyramid diagram on the left representing the physician-choice group shows longer light bars, indicating that the assay predicted more effective treatments than those prescribed by the physicians. In particular, 64.5% of subjects in the physician-choice group were treated with drugs that were not in accordance with the ChemoID assay prediction, and only 35.5% of subjects were treated with drugs that were found effective by the assay. In the ChemoID-guided group, 80.5% of subjects were treated with responsive drugs as predicted by the ChemoID assay, and 19.5% of the subjects were treated with less effective drugs due to their clinical health status (Table S2; Figure S1).

The distribution of cell kill predicted by the assay for the bulk of the tumor and the CSC assay of the drugs that were used to treat subjects in the physician-choice vs. the ChemoID-guided group is shown in Figure S4.

### Grade III/IV CRAEs association with chemotherapy in ITT analysis

The relative percentage of grade III/IV chemotherapy-related adverse effects (CRAEs) in ITT analysis was lower in the ChemoID assay-guided group (51%) vs. the physician-choice group (79%), with no unexpected neurological CRAEs or deaths due to CRAEs in either arm (Table S3). Toxicity side effects were consistent with the known safety profiles of the cytotoxic drugs used (i.e., no new safety concerns were observed). Thus, drugs predicted by the ChemoID assay did not cause more adverse effects than drugs chosen by physicians in the control group.

## Discussion

To improve the outcome of rGBM, it is critical to use chemotherapies that are effective against the CSCs as they are proven to drive tumor development and relapse. We conducted a randomized clinical trial using the ChemoID CSC assay to guide chemotherapy for rGBM treatment. It is worth noting that patients in both arms were treated with a regimen chosen from the same panel of chemotherapy medications, with one group using the patient-specific ChemoID test report to guide chemotherapy selection and the other relying on the physicians’ best judgment.

Planned interim efficacy analysis showed that the ChemoID-guided group’s mOS was 3.5 months longer than the physician-choice group’s (12.5 vs. 9 months) (Figure 3A). Additionally, mPFS was 6.6 months longer for patients receiving ChemoID assay-guided therapy compared with the patients treated using best-physician-choice chemotherapy (10.1 vs. 3.5 months) (Figure 3B).

In the ITT analysis, the study continued to meet its primary endpoint. The mOS was 4.5 months longer for the ChemoID-guided group compared with the physician-choice group (12 vs. 7.5 months) (Figure 3C). Additionally, mPFS was 3.2 months longer for patients receiving ChemoID assay-guided therapy compared with the patients treated using the best-physician-choice chemotherapy (6.5 vs. 3.3 months) (Figure 3D).

To confirm the validity of the data analysis using the HR method to summarize the difference in survival curves between the two arms as described in the approved study protocol, we also analyzed the data using RMST. By analyzing the 1- or 1.5-year RMST and CIs for each of our primary results, we found that subjects who received ChemoID-guided therapy had a significant survival advantage, which proves that using other robust statistical analysis methods, the survival differences between the two groups were statistically significant. The data support the use of the ChemoID assay for guiding chemotherapy selection for rGBM. The OS and PFS advantages observed for the ChemoID-guided group are not due to differences in prognostic variables, such as age, sex, performance status, *MGMT* promoter methylation status, or *IDH1*/*IDH2* gene status since all these variables were balanced during randomization between the two arms. Since the mutations of the IDH1/IDH2 genes could still cause differences in the results between the groups analyzed, we reanalyzed the data by removing subjects with mutant IDH1/IDH2 genes and found that the OS and PFS results remained unchanged.

The current SOC treatment protocol for GBM is a combination of surgical resection, radiotherapy, and concomitant TMZ chemotherapy followed by maintenance TMZ as demonstrated in the EORTC-NCIC trial.^1^^,^^2^ TMZ is a key component of standard therapy for patients both newly diagnosed and with rGBM. In newly diagnosed GBM, the addition of TMZ to radiotherapy resulted in 2.5 months of survival benefit, which led to TMZ being approved as a SOC.^2^^,^^25^ However, most patients with GBM experience recurrence and have a poor prognosis following the SOC treatment protocol. Treatment options after recurrence are limited, and no recent randomized clinical trial has demonstrated median survival longer than 10 months in rGBM,^10^^,^^26^ which is similar to the mOS of patients enrolled in the control group (best physician choice) of our trial. This randomized study demonstrates that using the ChemoID CLIA-certified clinical laboratory assay to select effective cytotoxic therapies for the treatment of rGBM is a promising and cost-effective strategy for increasing the survival of patients.

Of note, the use of anti-CSC-guided therapy resulted in a 3.5-month mOS advantage when compared with the control group in the population evaluated at the interim efficacy analysis and a 4.5-month mOS advantage in the ITT population. A significant difference was observed in the risk of death between the two groups. More participants survived in the ChemoID assay-guided group compared with the non-guided group at 6, 9, and 12 months, demonstrating that patients with rGBM derive a survival benefit from treatment with CSC-directed therapy.

In conclusion, the ChemoID assay was developed as an actionable tool for physicians to individualize cancer treatment by selecting the most effective therapies against CSCs from a panel of cytotoxic agents that are common and affordable for cancer patients. Treatments with more expensive targeted anti-cancer drugs and immunotherapies are not always feasible due to socioeconomic and health disparity issues in the US and around the world. Although there are newer targeted therapies, our clinical trial focused on screening SOC chemotherapies that are routinely covered and used by community oncologists globally. The results of our study highlight the clinical effectiveness of a personalized approach to treatment. The ability of the ChemoID assay to personalize chemotherapy selection is a promising way to provide more affordable treatment for patients with rGBM. The ChemoID assay is versatile, allowing it to be expanded to include other new agents. We anticipate personalized anti-cancer therapy targeting CSCs will be included sooner in the treatment plan, eliminating ineffective treatments and allowing patients to gain the greatest therapeutic benefit possible.

### Limitations of the study

Although this study provides good treatment options for patients with rGBM, some potential limitations should be noted. For example, ChemoID is a functional assay limited by the availability of viable tumor tissue samples. Our study only included rGBM subjects who underwent surgical resection or biopsy. Subjects with inoperable tumors or who were in poor health were not participants in our study. Future studies should incorporate patients with newly diagnosed *MGMT* unmethylated GBM, who would benefit from assay-guided intervention. In addition, further studies should investigate the use of genomic assays with this functional assay in larger cohorts for guiding treatment.

## STAR★Methods

### Key resources table

**Table:** 

REAGENT or RESOURCE	SOURCE	IDENTIFIER
**Biological samples**		

Glioblastoma tissue samples	This Study	N/A

**Chemicals, peptides, and recombinant proteins**		

Carboplatin solution for Injection	Hospira Inc.	00703-4244-01
Carmustine (BCNU)	Sigma Chemical Company	C0400-25MG
Etoposide Solution for Injection - 20 mg/ml	Pfizer, Inc.	16729-01114-31
Imatinib Mesylate (STI571)	Selleck Chemicals, LLC	220127-57-1
Irinotecan Hydrochloride Injection	Pfizer, Inc.	0009-7529-03
Lomustine (CCNU)	Sigma Chemical Company	L5918-100MG
Procarbazine (hydrochloride)	Cayman Chemical Company	16133
Temozolomide	Cayman Chemical Company	14163
Vincristine Sulfate Injection	Hospira, A Pfizer Company	61703–0309
Accutase	MP Biomedical LLC	91000449
Amphotericin-B	Thermo Fisher Scientific (Gibco)	15290018
Dimethyl Sulfoxide (DMSO)	Alfa Aesar	67-68-5
Ethanol 200 Proof (Absolute)	Aldrich Chemical Company	64-17-5
Fetal Bovine Serum: Characterized, US-Sourced	HyClone Laboratories, Inc.	SH3087901
Gentamycin Sulfate	Acros Organics	AC61398-0010
Penicillin-Streptomycin	Thermo Fisher Scientific (Gibco)	MT30001CI
Phosphate Buffered Saline (PBS) Tablets	Life Technologies Corporation	18912014
RPMI-1640	HyClone Laboratories, Inc.	SH30027LS
Sterile Water	Cardinal Health	50-487-337
Thiazolyl Blue Tetrazolium Bromide (MTT)	Sigma Chemical Company	ICN 102227-01
Trypan Blue	Acros Organics	AC189351000

**Experimental models: Cell lines**		

Glioblastoma primary cell lines	This study	N/A
Glioblastoma Cancer Stem Cells	This study	N/A

**Software and algorithms**		

Cell Counting Software Nexcelom	Nexcelom	Cellometer Mini
Softmax-Pro Molecular Devices	Molecular Devices	Softmax Pro 7.0.3
Encompass ChemoID Data Analysis	In-House Programming	Microsoft Office Excel 2016

**Other**		

Class II BioSafety Cabinet	Kewaunee	INT-4000
SpectraMax 340PC 384 Absorbance Microplate Reader	Molecular Devices	LNR06595 (SN), LNR066596 (SN)
Cellometer Mini Cell Counter	Nexcelom	SKU: Cellometer Mini
Water Bath	VWR	28487 05X
Sorvall Legend XTR Centrifuge	Thermo Fisher Scientific	50119927–4
HERAcell CO_2_ Incubator	Thermo Fisher Scientific	50115191B
Model 900 Series Ultra Low −80 Freezer	Thermo Fisher Scientific	24020/FR-2145
Revco Laboratory Freezer −20	Thermo Fisher Scientific	UFP430A
Rotating 3D-cell culture bioreactor	Cordgenics	CG0001
XS603S Analytical Balance	Mettler Toledo	1126402591 (SN)
Revco Laboratory Refrigerator	Thermo Fisher Scientific	RGL5004
Zeiss Cell Culture Inverted Light Microscope	Thermo Fisher Scientific	491206-0011-000
Sterile Single Use Serological Pipettes (5mL, 10mL, 25mL)	Thermo Fisher Scientific	1367811E, 1367811
Sterile Micropipette Tips (10μl, 20ul, 100ul, 200ul, 1000ul)	Thermo Fisher Scientific	02-707-441, 02-707-402, 02-707-419, 02-100-503)
Tissue Culture Treated 10cc Petri Dishes (Biolite)	Thermo Fisher Scientific	12556002
Tissue Culture Treated 96-well Flat Bottom Microplates	Thermo Fisher Scientific	12-556-008
Sterile 1.5mL Microcentrifuge Tubes	Cardinal Health	02-681-258
Sterile Conical Tubes (10mL, 50mL)	Thermo Fisher Scientific	05-527-90, 06-443-19
Sterile Scalpels, Single Use	Cardinal Health	03-025-678
Sterile Single Use Reservoir 25mL	Thermo Fisher Scientific	14222399
Parafilm	Thermo Fisher Scientific	P1150-2

### Resource availability

#### Lead contact

Further information and requests for resources and reagents should be directed to and will be fulfilled by the lead contact, Pier Paolo Claudio (claudio@cordgenics.com).

#### Materials availability

This study did not generate new unique reagents.

### Experimental model and subject details

#### Patients

123 patients affected by recurrent GBM or grade III glioma were screened in this parallel-group randomized controlled clinical trial at 13 clinical sites across the US over a period of three years and assessed for inclusion and exclusion criteria to participate in the study (Table S4). Subjects eligible to participate in the study were men and women and members of all ethnic groups, at least 18 years old at the time of enrollment, who were affected by a surgically resectable first recurrence of grade-III glioma, and grade-IV recurrent glioblastoma (GBM), inclusive of gliosarcoma. In all cases, the diagnosis had to be confirmed by a pathologist according to the 2016-WHO classification of brain tumors.^27^ Even though the primary inclusion requirement was the presence of recurrent grade-III glioma or grade-IV GBM, our trial only enrolled participants with 2016-WHO confirmed recurrent grade-IV glioma (rGBM). At the time that this clinical trial started enrolling patients, the WHO classification for brain tumors was in accordance with the 2016 guidelines. In August 2021, the updated 2021-WHO guidelines were published,^28^ while our trial was completed in November 2021. All patients had already received first-line treatment with surgery, radiotherapy, and TMZ at the time of enrollment. Patients were excluded if they had another active malignancy or were receiving any other tumor-directed therapy (e.g., tumor treating fields device).

78 recurrent GBM patients were enrolled in the study (Consort Diagram – Figure 1). Registered participants provided an MRI (or CT if the patient was unable to have an MRI performed) of the brain with and without contrast within 14 days of the screening visit. Blood samples were drawn as per standard-of-care and used to confirm eligibility based on clinical laboratory parameters. Female participants had a urine or serum pregnancy test.

Subjects underwent surgical resection and biopsy. For histopathology confirmation and diagnosis of GBM, MGMT gene methylation status, and IDH-1/2 status, fresh tissue tumor biopsies from rGBM patients were sent to the sites’ hospital pathology lab for processing. The central ChemoID laboratory conducted the drug response assay using a second portion of the fresh biopsies. In accordance with CLIA and CAP requirements, samples were shipped to the ChemoID laboratory utilizing a secure FedEx overnight shipping container for clinical specimens. All recurrent GBM patients who registered for the trial underwent the ChemoID assay and were randomly assigned by the sites’ coordinators to a study group using a computer-generated algorithm (in REDCap).

The patients were treated either with the standard of care chemotherapy chosen by the physician (ARM 1) or treatment directed by the ChemoID assay (ARM 2), depending on the randomly allocated study group. Data collection was performed by a REDCap electronic data capture application software.

Chemotherapy medications were administered in accordance with the trial group to which each patient was assigned. Over the course of treatment, assessments of adverse events and drug compliance were made. Enrollment in the trial began in May 2018 after the lead institution completed the trial start-up procedures. Our trial was registered on ClinicalTrials.gov with Identifier NCT03632135 in August 2018 after all clinical research agreements with participating institutions were completed, even though the FDA’s Final Rule for Clinical Trials Registration considered our trial registration to be optional (42 CFR Part 11). A secure password-protected REDCap web portal was available for authorized study coordinators to input clinical trial data. Patients were blinded to randomization group assignment. Unblinding of test results was not permitted. Investigators and trial personnel were not aware of ChemoID test results for patients in the physician-choice therapy group until the end of the study. Investigators and trial personnel received the ChemoID lab test results **only** for subjects assigned to the assay-guided group. Data monitoring and analysis of results were conducted by independent statistical services and a data manager. Patient safety and adverse events (AEs) during the trial were monitored by an independent data safety monitoring board (DSMB).

The study protocol was approved by the Western Institutional Review Board (WIRB) and each of the independent ethics committees of the participating institution. This study was conducted in accordance with the Declaration of Helsinki and Good Clinical Practice (GCP) requirements described in the current revision of the International Conference on Harmonisation of Technical Requirements of Pharmaceuticals for Human Use (ICH) Guidelines and all applicable regulations, including current United States Code of Federal Regulations (CFR), Title 21, Parts 11, 50, 54, 56, and 312 and Title 45, Part 164. The IRB reviewed and approved the site’s informed consent form (ICF), and any other written information that was used for patient recruitment. All patients signed the informed consent before enrollment.

### Method details

#### Patient treatment

Lead investigators agreed on the cytotoxic chemotherapies used in the trial, and the health insurance plans covered them, thus study participants incurred no additional medical bills. The regimens and doses tested by the ChemoID drug response assay were the same as the ones that could be chosen by the physicians for patients enrolled in the control arm (Table S1).

Patients received 1 of 14 cytotoxic chemotherapy regimens either chosen by the physician or guided by the ChemoID assay test report (Figure 2). Physicians and investigators were not provided with ChemoID test reports for patients randomized to the physician-choice control arm. Regardless of which arm the patient was assigned to, the ChemoID test was performed on all subjects so that retrospective analysis could be conducted on patients randomly assigned to the Physician-choice group.

The assay-guided group received the regimen that killed the most cancer stem cells and the bulk of the tumor. The treatment given to subjects in the control group was chosen from the same list of chemotherapies tested by the assay (Table S1), based on the treating physician’s best empirical judgment. The number of chemotherapy drug cycles in both trial arms was determined at the treating physician’s discretion. Patients were, however, treated for a minimum of 4 cycles and continued to receive treatment until unacceptable toxicity, hospice or death, or consent withdrawal.

For patients in the assay-guided group, in the event of unacceptable toxicity or progression, treatment was changed to the next best chemotherapeutic drug or combination based on the ChemoID assay report. In cases in which the assay predicted more than one high-cell kill drug, for patients randomized to the assay-guided arm, the protocol gave the physicians the ability to choose a treatment among the high-cell kill drugs based on the ChemoID assay report that would benefit the patient, considering the patient’s general health status.

Bevacizumab is not expected to improve overall survival^4^ and was permitted in the clinical trial. If indicated, it was started at least 4 weeks following the craniotomy or biopsy, when the wound had healed well without any drainage or cellulitis.

In the absence of treatment delays due to the presence of adverse event(s), treatment continued as specified in the above treatment modality sections or until one of the following criteria was applicable: hospice or patients’ death, intercurrent illness that prevents further administration of treatment, unacceptable adverse event(s), patient decides to withdraw consent for participation in the study, or general or specific changes in the patient’s condition render the patient unacceptable for further treatment in the judgment of the investigator.

#### ARM 1 - Physician’s choice of chemotherapy regimens

Patients randomized to the physician-choice chemotherapy arm were treated with one of the regimens from the list of chemotherapies specified per the investigator’s discretion. Patients received treatment as per standard practice and continued on the treatment until hospice or as per investigator’s discretion if having continued response (SD, PR, or CR) and/or clinical benefit. The number of cycles of therapy administered as clinically appropriate was based on the health status, although it was recommended that patients should receive at least 4 cycles of therapy.

#### ARM 2 – ChemoID-guided drug response assay chemotherapy regimens

The physician selected a treatment regimen based on ChemoID drug response assay results on cancer stem cells (CSC) and the bulk of tumor cells. Ideally, the regimen with the highest percentage cell-kill for cancer stem cells and the bulk of tumor combined was used; however, the physician had the flexibility to choose the best regimen according to anticipated patient tolerability. The regimens tested by the ChemoID drug response assay are the same as the ones that can be chosen by the Physician for patients enrolled in Arm 1.

#### Patient follow-up

Participants were followed for three years according to standard-of-care intervals by neurologic and neurosurgical clinical assessments or until death. At 6 and 12 months after planned Visit 24 there was a phone call to assess survival status.

Participants were assessed at follow-up visits following standard-of-care treatments and chemotherapy drugs were dispensed according to groups and cohorts. Drug compliance and adverse event assessment were performed. Lab work and brain imaging were collected at visits as per standard-of-care.

The outpatient visits window was ± 7–14 days from the intended date of the visit. Follow-up visits consisted of a clinical evaluation with particular attention to neurological function, seizures, and corticosteroid use as per standard-of-care management of the disease. Laboratory tests of blood counts, glucose levels, and blood count, liver function tests indicated if the participant was receiving chemotherapy, corticosteroids, and anti-epileptic drugs.

CT scan or MRI was performed as standard of care for the entire time patients are in the trial, and at any other time if clinically indicated based on symptoms or physical signs suggestive of progressive disease. Imaging assessments were discontinued once a patient was off of the clinical trial, hospice, or death. Patients were followed for overall survival during the clinical trial.

Participants were followed according to SOC intervals determined by neurologic and neurosurgical clinical assessments preferably with brain MRI scans pre- and post-intravenous gadolinium contrast unless the patient had a contraindication to gadolinium contrast then non-contrast brain MRI was obtained and or CT-scans of the brain pre and post intravenous contrast or without contrast, if the patient had a contraindication to CT intravenous contrast such as severe allergy and/or renal dysfunction.

During treatment, adverse events were assessed according to National Cancer Institute Common Terminology Criteria for Adverse Events (version 5.0). Post-chemotherapy, patients had follow-up visits every 3 months, during which neurological function and corticosteroid levels were assessed and contrast-enhanced MRIs were performed (in addition to the post-surgery/baseline MRI).

Response to chemotherapy was evaluated according to the 2D Response Assessment in Neuro-Oncology (RANO) criteria, in which in addition to contrast enhancement, tumor extension on T2-and fluid-attenuated inversion recovery (FLAIR)-weighted MRI are evaluated.^29^^,^^30^

Tumor assessments were performed by an independent neuro-radiology service composed of 2 readers and a third senior reader for adjudication of disagreements. All neuro-radiologists were blinded to groups and/or treatment assignments throughout the trial to determine the earliest time of progression independent of the impressions of the treating physicians to avoid bias.

A record of all concomitant (OTC & prescription) taken 30 days prior to the screening visit through study termination was taken.

#### Clinical trial monitoring evaluations and measurements

From the medical chart (paper or electronic) this additional data was collected: age, gender, weight, pathology report, steroid and other medication doses over the course of treatment, ChemoID test results, MGMT gene methylation status, IDH-1 mutation status, chemotherapy regimens including doses, all brain imaging including but not limited to DICOM images of MRI and or CT scans, clinical assessment of disease at baseline and during the course of therapy from neuro-oncologic progress notes, Health-Related Quality of Life (HRQOL) questionnaires addressing physical, psychological, emotional, and social issues.

#### Risk assessment

The ChemoID assay was classified as a non-significant risk assay for patients by the ethics committees. The current study utilized only sample specimens obtained by established procedures that patients undergo routinely for the treatment of his/her recurrent cancer; there was no additional risk to the patient. Tissue for this study was obtained by patient consent and after it was assured that there was adequate tissue for routine histologic analysis. At no time tissue was obtained solely for carrying out the ChemoID assay. No investigational agents were included in the trial. There were no greater than minimal risks associated with this study since all chemotherapy drugs used were FDA-approved for the treatment of recurrent 2016-WHO grade III or IV gliomas.

#### Patient tumor sample collection and processing

Inclusion criteria included patients 18 and older with first-recurrence of grade III or grade IV glioma (according to the 2016-WHO guideline classification),^27^ who were able to provide fresh tissue of the primary lesion. After informed consent, eligible participants underwent surgical resection or stereotactic biopsy of the tumor as per standard of care. Fresh tissue biopsy samples were collected in the operating room under sterile conditions and divided into two parts. One part of the biopsy was sent for testing in a sterile vial containing RPMI transportation medium at room temperature via overnight FedEx clinical pack to the ChemoID laboratory. Upon arrival, patients’ identifiers were recorded, and the tissue was triaged for the growth of bacteria and yeast/fungi and accepted at the ChemoID laboratory. The assay used in this study to guide treatment was performed by an independent hospital pathology laboratory regulated by the Centers for Medicare & Medicaid Services (CMS), which oversees all laboratory testing performed on humans in the U.S. through the Clinical Laboratory Improvement Amendments (CLIA) guidelines. The second portion of the biopsy was placed in a 10% formaldehyde solution and sent to the local pathology lab for histopathological confirmation to satisfy the main inclusion criterion. Tissue samples were also evaluated for methylation of the *MGMT* gene promoter and *IDH1*/*IDH2* gene mutation status. Post-surgery/biopsy, patients received a baseline contrast-enhanced brain MRI.

#### Isolation of cancer cells from tumor biopsies

To generate the primary tumor cell cultures, the fresh brain tumor tissue from surgical biopsies was minced using sterile scalpel blades and gently dissociated in a biosafety cabinet using 0.025% trypsin solution at 37°C for 10 min with gentle agitation and intermittent resuspension. Dissociated tumor cells were plated in RPMI with 20% FBS, 1% Penicillin/Streptomycin, Gentamycin Sulfate (complete media) in sterile plastic Petri dishes in the presence of residual tumor tissues and incubated at 37°C humid tissue culture incubator in the presence of 5% CO_2_. Primary cancer cells were passaged to confluency and sub-cultured in complete media in additional sterile plastic Petri dishes.

#### Enrichment of cancer stem cells (CSCs)

Patient-derived CSC cultures were obtained as previously described in.^20^^,^^21^^,^^31^^,^^32^ The CSCs were enriched from the primary tumor cell cultures by loading a 3D cell culture rotating bioreactor (Cordgenics) with a volume of 40 mL and a gas-permeable membrane that allows for gas exchange where cells will aggregate in suspension to form spheroids or cell aggregates in the absence of shear forces.^31^^,^^32^ The 3D-suspension cell culture rotating bioreactor, provides the capability to control the movement of air bubbles and removes them from the bioreactor without degrading the low-shear culture environment or the suspended three-dimensional tissue assemblies. This provides unparalleled control over the locations of cells and tissues within its bioreactor vessel during operation and sampling. Both the low-shear suspension of cells and control of the locations of cells and air bubbles are affected by means of the hydrodynamic force created by the flow within the vessel and fluid drag along the surface of the viscous spinner. A gas-permeable membrane connected to the base of the vessel enables the exchange of gas between the tissue culture medium in the vessel and an incubator environment in which the vessel is placed. The presence of a conic spinner on the axis of rotation of the cell culture rotating bioreactor enables the simultaneous creation of a low-shear culture environment and the “herding” of suspended cells and tissue assemblies, which is responsible for the CSCs' selective growth. A rotation rate of 15–25 rpm was estimated to have average sheer values of 0.001 dyn per square centimeter, which is the rate at which medium-large, three-dimensional, tissue-like suspended growth assemblies have been successful. This 3D-suspension cell culture rotating bioreactor configuration was shown previously to select and enrich 15-fold cancer cell cultures expressing markers of CSCs such as CD133 among others.^31^ Other conventional bioreactors rely instead on agitation to suspend cells and attachment materials and to facilitate the mass transfer required for the growth of cells and tissue assemblies. However, the shear force generated by agitation can affect cell-cell interactions and degrade three-dimensional tissue development.

CSCs from primary cancer cells (bulk of the tumor cells) were enriched by loading 2x10ˆ6 bulk of tumor cells into the bioreactor and culturing them for 7- days in RPMI media in the absence of growth factors.^20^^,^^21^^,^^22^ The rotating bioreactor was maintained in an incubator with constant CO_2_, temperature, 20% airflow, and at 20–25 rpm rotation speed. Validation of the bioreactor-enrichment of CSCs from GBM biopsies was achieved by immunophenotyping bulk of tumor and CSCs expression of CD133, CD24, and CD44 using flow cytometric analyses, and by xenografting the CSCs in immune-deficient mice in a limiting dilution assay to verify their tumor-initiating capacity *in vivo* as shown in Figure s5 and Table S5.^20^^,^^31^^,^^32^

#### Assessment of CSCs' and bulk of the tumor chemotherapy response

Treatments with anti-cancer drugs and sensitivity tests were performed as described previously in.^20^^,^^21^^,^^31^^,^^32^ The bulk of tumor cells and CSCs were counted using trypan blue exclusion to determine cell viability and cell number prior to chemosensitivity testing using a Cellometer mini automated cell counter.

96-well plates are seeded in RPMI-1640 with 10% FBS, penicillin and streptomycin with a minimum of 20,000 individual tumor cells per regimen of bulk tumor cells or CSCs in 5 replicas and incubated at 37°C in a 5% CO_2_ incubator. After 24 h from plating, clinical-grade chemotherapy drugs were added alone or in combination for 1-h exposure at concentrations that do not exceed the serum C[max] described in pharmacokinetic (PK) studies, including the clinical dose. Three concentrations of each chemotherapy treatment were prepared by serial dilution. Each concentration was added to five replicate wells on the microtiter plate. Additionally, three replicated wells (control 1 = no treatment) and three replicated wells (control 2 = equal amount of solvent) were associated with each treatment.

After the 1-h exposure, the treatment media containing the various chemotherapies were removed and replaced with fresh media. MTT assay was performed 24 h following chemotherapy treatment to assess cell survival as previously described.^20^^,^^21^^,^^22^

Inhibition of bulk tumor cells and CSCs survival was measured for each concentration (average counts in five replicates ±SE) of a given treatment (for a total of 15–18 different treatments per patient). Survival of tumor cells at each concentration was calculated as compared to control-2 and the overall percent of the bulk of tumor cells and CSCs killed was calculated for each treatment as the primary measures of potential therapy efficacy.

#### Reporting of the assay

Percent survival (potential therapeutic efficacy) was calculated relative to appropriate negative and positive controls for each treatment. Efficacy and resistance of each drug and combinations were reported on the ChemoID assay results as a continuous number from <10% to 100% cell-kill as previously.^20^^,^^21^^,^^22^

### Quantification and statistical analysis

All statistical analyses followed the plan specified in the protocol with no deviations and were completed using Stata v17.1 (StataCorp) by independent biostatisticians. Two prespecified interim efficacy analyses were planned *a priori* at 35 and 70 death events (1/3 and 2/3 of the full target sample size number of events). The hazard ratio (HR) for ChemoID-guided: Physician-choice to declare efficacy was <0.55 at all three-time points. The Lan-DeMets alpha spending approach was utilized to determine P-value stopping criteria for the two prespecified interim efficacy analyses. Efficacy could be declared at the first analysis if HR was <0.55 with an associated P-value <0.0167.

Baseline characteristics were compared using t-tests or Mann-Whitney U tests, as appropriate, for continuous and ordinal variables and Fisher’s exact tests for categorical variables. Kaplan-Meier curves were constructed using established methods, and median survival was calculated from these curves. Primary OS and secondary (PFS) HRs were constructed using Cox proportional hazard models with baseline HRs stratified by clinical site. Variance component estimation was performed via bootstrap resampling using 1000 bootstrapped replicates. Specific time-point survival probabilities (6, 9, and 12 months) were calculated using marginal probabilities obtained via logistic regression models, and associated odds ratios were reported. Additionally, an exploratory analysis was performed using a 1- or 1.5-year restricted mean survival time (RMST) to quantify the treatment effects.

#### Baseline data

As per protocol, initial analyses involved data cleaning, variable development, and exploratory data analyses. We used standard summaries to describe baseline characteristic distributions in terms of centrality, spread, shape, and possible outliers by arm, cohort, and treatment group. Graphical explorations emphasized the examination of the nature and extent of potential nonlinear relationships on the appropriate modeling scale (e.g. natural, log, logit, etc.)

#### Efficacy analysis

The primary analysis was based on an intention-to-treat approach and included all subjects randomized at baseline. The primary efficacy outcome was overall survival (OS) in months. This outcome was compared between patients randomized to ChemoID-guided chemotherapy) versus standard of care. OS comparisons were examined using Cox Proportional Hazard Models for Overall Survival with baseline hazards stratified by site and medians were compared between treatment arms. Models examining adjustments for sex, race, age, and tumor stage were constructed, as well as for moderating effects of these variables (subpopulation investigations).

Secondary analyses included logistic regression models for Overall and Progression-Free Survival at 4, 6, 9, and 12 months, Cox Proportional Hazard Models for Progression-Free Survival in months, Generalized Linear Models (GLMs) for analyses on objective tumor response (RANO), and Health-Related Quality of Life (HRQOL). Generalized Linear Mixed Models (GLMMs) were used for analyses of changes in any additional repeated outcome measures to incorporate within-person associations and examine distributions of participant-specific declines. Huber-White robust standard errors were used, and multiple variance structures were investigated to examine the sensitivity of primary analyses to the choice of association model. Shared Parameter Models (SPM) were used to examine any potential informative missing data effects.

#### Interim analysis

We performed a predetermined interim analysis as per protocol. From the clinical trial protocol, two interim analyses based on the alpha spending approach of Lan and DeMets could be performed. One would be performed whenever 35 patients had passed away. If both the observed HR was less than or equal to 0.55 and its related p-value was less than 0.0167, the trial may be stopped for efficacy at this point. If the trial continued, the second interim analysis would take place after 70 people had expired. If the observed HR was less than or equal to 0.55 and its corresponding p-value was lower than or equal to 0.0218, the trial could be stopped for efficacy at this point.

## Data Availability

• **Data**: The study protocol and statistical analysis plan will be available from the lead contact author upon request. The data that support the findings of this study are not openly available due to patient privacy, ethical, and legal issues. The de-identified participants’ data that underlie the results reported in this article, will be made available upon reasonable request to investigators whose proposals for the use of the data have been approved by an independent review committee. Proposals may be submitted to the corresponding author beginning 12 months up to 18 months from the publication date. • **Code**: This paper does not report original code. • **General statement**: Any additional information required to reanalyze the data reported in this work is available from the lead contact upon request.

## Supplemental information

Document S1. Figures S1–S5 and Tables S1–S5

Document S2. Article plus supplemental information
