# Supplementary material for: Cancer stem cell assay-guided chemotherapy improves survival of patients with recurrent glioblastoma in a randomized trial
Source: Cell Rep Med. 2023 May 2;4(5):101025. doi: 10.1016/j.xcrm.2023.101025 (PMC10213810; doi:10.1016/j.xcrm.2023.101025)
Supplement: Document S1. Figures S1–S5 and Tables S1–S5 [file mmc1.pdf]

Cell Reports Medicine, Volume *4*

Supplemental information

Cancer stem cell assay-guided chemotherapy improves survival of patients with recurrent glioblastoma in a randomized trial

Tulika Ranjan, Soma Sengupta, Michael J. Glantz, Richard M. Green, Alexander Yu, Dawit Aregawi, Rekha Chaudhary, Ricky Chen, Mario Zuccarello, Christine Lu-Emerson, Hugh D. Moulding, Neil Belman, Jon Glass, Aaron Mammoser, Mark Anderson, Jagan Valluri, Nicholas Marko, Jason Schroeder, Steven Jubelirer, Frances Chow, Pier Paolo Claudio, Anthony M. Alberico, Seth T. Lirette, Krista L. Denning, and Candace M. Howard

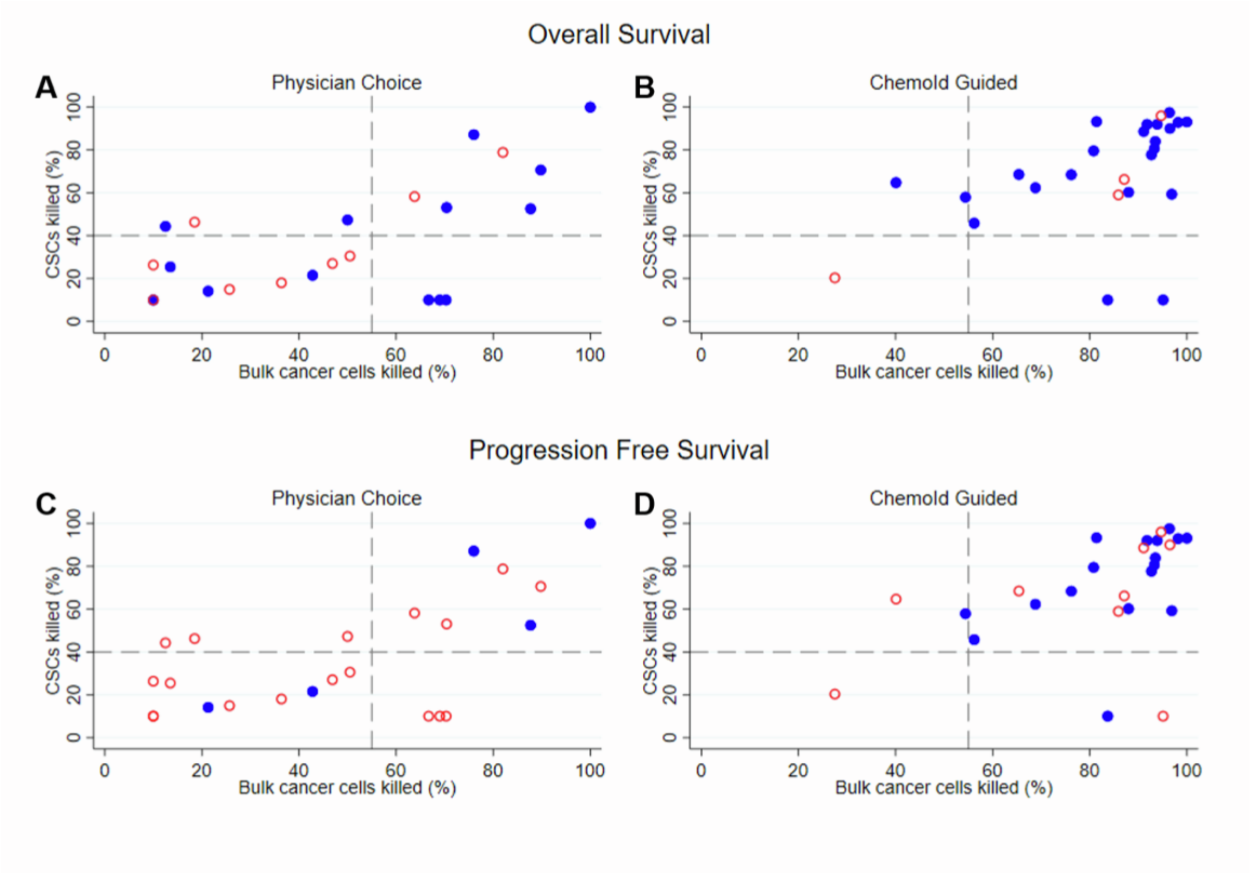

Supplemental Figure 1. Patient' OS and PFS correlated with the cell kill of drugs used during treatment as per the ChemoID test report, related to figure 3

A) Quadrant diagrams of the associative analysis of cell kill percentages (bulk tumor cell and CSCs) vs patient OS at 6-months post-randomization. Open-red circles, participants who had died; solid blue circles, participants surviving.

B) Quadrant diagrams of the associative analysis of cell kill percentages (bulk tumor cell and CSC) vs patient PFS at 6-months post-randomization. Open red circles, participants who had progressed; solid blue circles, participants who had not progressed.

Referent lines, 40% for CSCs and 55% for bulk tumor cells indicate the optimal thresholds from the logistic regression models.

A

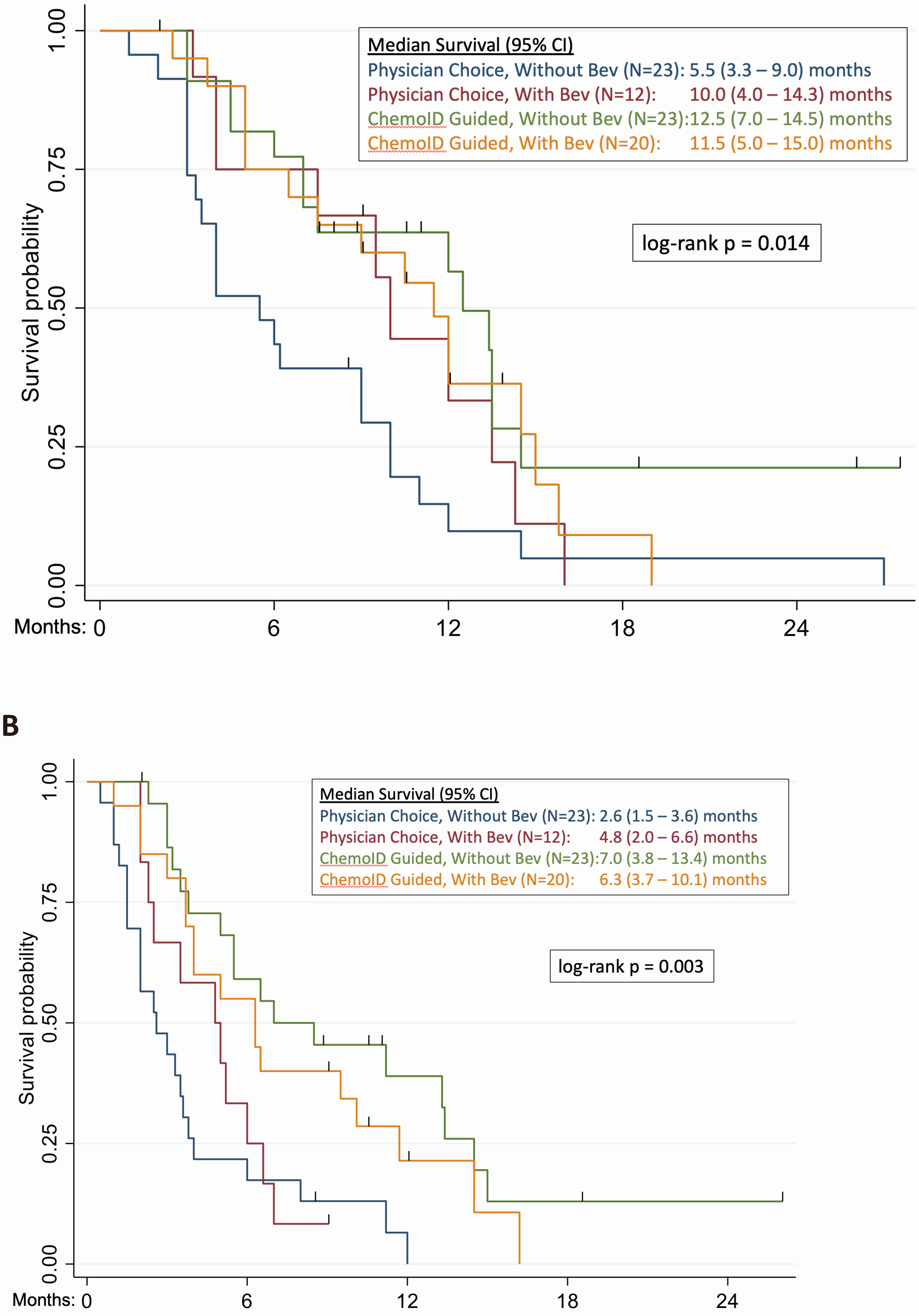

B

Supplemental Figure 2. Kaplan Meier plots of OS and PFS stratified by the use of Bevacizumab during chemotherapy treatment, related to figure 3

A) ITT Kaplan Meier analysis of OS

B) ITT Kaplan Meier analysis of Progression Free Survival (PFS)

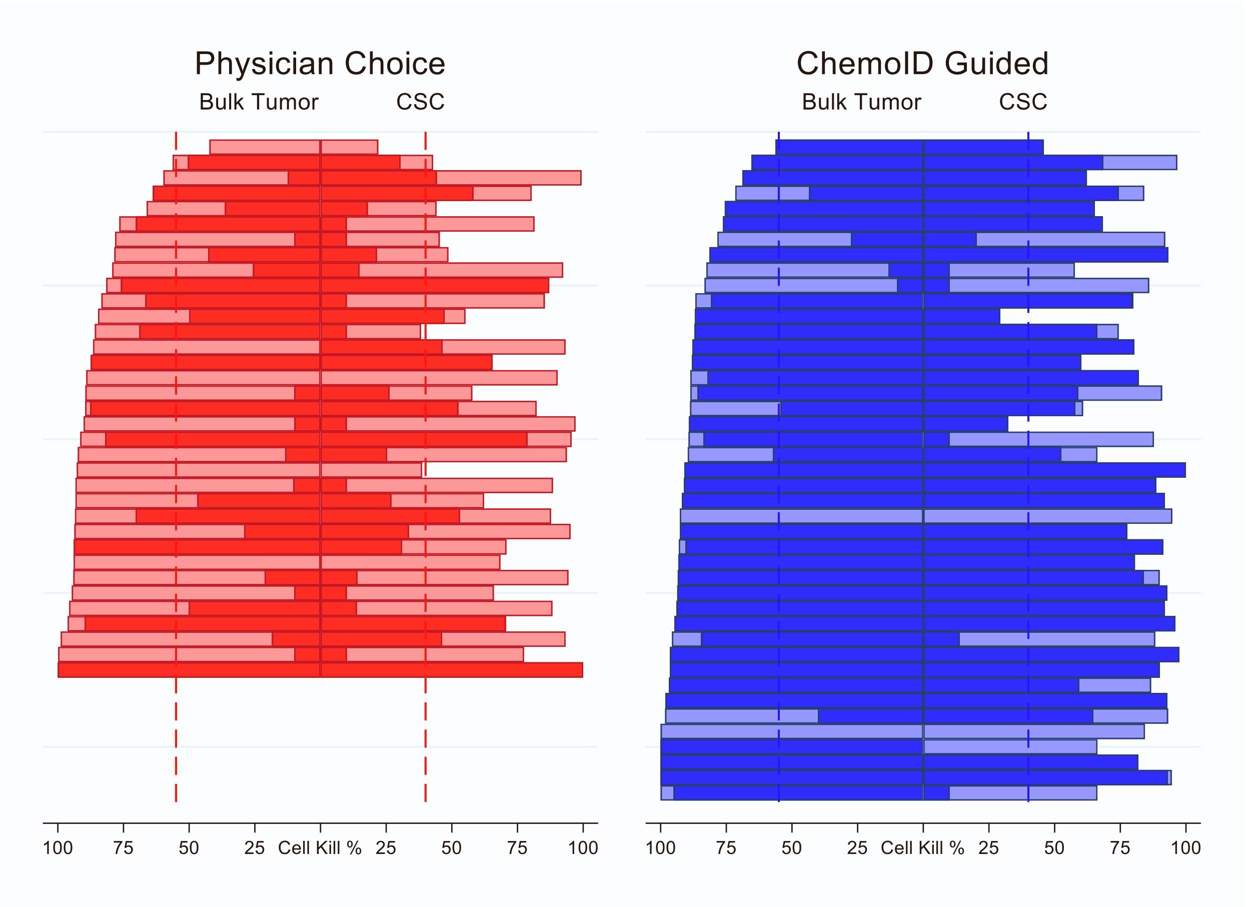

Supplemental Figure 3. Comparison of the most effective drug found by the ChemoID assay from a panel of various chemotherapies versus the actual kill percentages of the chemotherapy treatment used in Physician Choice and ChemoID-Guided groups for each patient, related to figure 3

Pyramid plot comparing CSC and bulk tests for each patient, showing the percent cell kill for the most e ective drug as predicted by the ChemoID assay and the actual percent cell kill of the chemotherapy regimen utilized. ChemoID-identified optimal therapies with the highest cell kill are shown in light colors. Therapies used and their cell kill are shown in dark colors, with each row of the pyramid corresponding to results for a single patient. When the light bar is longer than the dark bar, the ChemoID assay identified a more optimal therapy than the one that was administered.

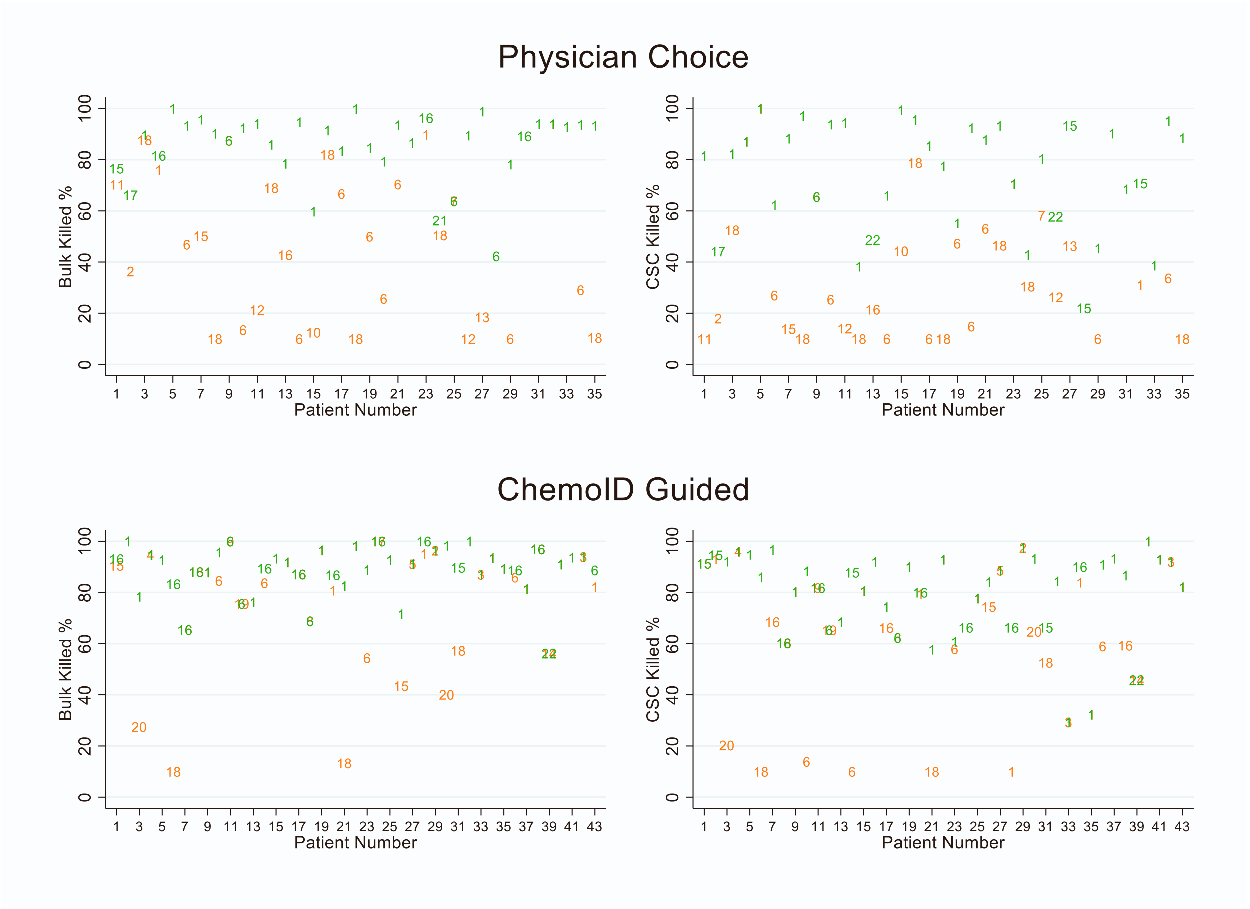

Supplemental Figure 4. Cell-kill diagram for the panel of tested chemotherapy and their combinations across all subjects with patient numbers on the x-axis and drug observed cell-kill on the y-axis, related to figure 3

Green numbers represent the ChemoID-identified best drug(s) and orange numbers are the drugs used for therapy. 1: BCNU; 2: BCNU, Carboplatin; 3: BCNU, Etoposide; 4: BCNU, Imatinib; 5: BCNU, Imatinib, Etoposide; 6: CCNU; 7: CCNU, Etoposide; 8: CCNU, Etoposide, TMZ, Imatinib; 9: CCNU, TMZ, Imatinib, Procarbazine, Etoposide; 10: Carboplatin, Irinotecan; 11: Carboplatin, TMZ, Irinotecan; 12: Etoposide; 13: Etoposide, Carboplatin; 14: Etoposide, Vincristine; 15: Imatinib; 16: Imatinib, TMZ; 17: Procarbazine, CCNU, Vincristine; 18: TMZ; 19: TMZ, CCNU; 20: TMZ, Carboplatin; 21: Irinotecan; 22: Vincristine.

A)

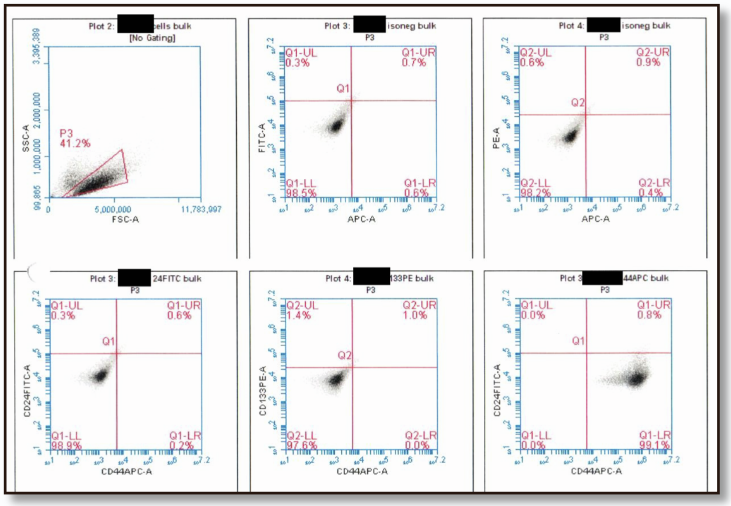

B)

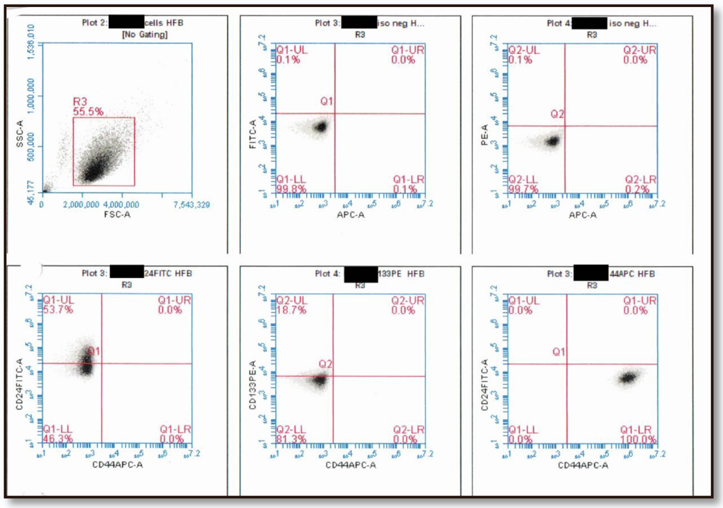

Supplemental Figure 5. Example of flow cytometric analysis of CD133, CD44, and CD24 expression in a patient-derived primary GBM cell line (Bulk of Tumor - Baseline) and bioreactor-enriched CSCs, related to STAR methods

A) Bulk of the Tumor - baseline. CD133 1.4%; CD24 0.3%; CD44 99.1%
B) Bioreactor-enriched CSCs. CD133 18.7%; CD24 53.7%; CD44 100%

Supplemental Table 1. List of Chemotherapeutic Agents and Combinations with doses, related to STAR methods

	**Single drugs**	**Dose**
**1**	Carboplatin	350 mg/m2 or 4 AUC
**2**	Irinotecan	125 mg/m2
**3**	Etoposide	50 mg/m2
**4**	BCNU	100 mg/m2
**5**	CCNU	100 mg/m2
**6**	Temozolomide	150-200 mg/m2
**7**	Procarbazine	60 mg/m2
**8**	Vincristine	1.4 mg/m2
**9**	Imatinib	400 mg
	**Drug combinations**	**Dose**
**1**	Procarbazine	60 mg/m2
	CCNU	100 mg/m2
	Vincristine	1.4 mg/m2
**2**	Carboplatin	350 mg/m2 or 4 AUC
	Irinotecan	125 mg/m2
**3**	Carboplatin	350 mg/m2 or 4 AUC
	Etoposide	50 mg/m2
**4**	Temozolomide	50 mg/m2
	Etoposide	50 mg/m2
**5**	Temozolomide	50 mg/m2
	Imatinib	200 mg

Supplemental Table 2. The accordance between the ChemoID assay prediction and treatment administered, related to figure 3

	Physician-choice group	ChemoID-guided group
**Accordance of assay prediction with regimen used**		
**No**	64.5%	19.5%
**Yes**	35.5%	80.5%

Supplemental Table 3. Treatment-related AE observed, related to figure 3

Adverse events	Physician-choice group			ChemoID-guided group		
	AE grades 1-4*	AE grades 1-2*	AE grades 3 & 4*	AE grades 1-4*	AE grades 1-2*	AE grades 3 & 4*
**All, No.**	92	50	42	87	44	43
**Chemotherapy - related, No. (%)**	54/92 (59%)	25/50 (50%)	33/42 (79%)	46/87 (53%)	19/44 (43%)	22/43 (51%)
* Grades: 1, mild; 2, moderate; 3, severe; 4, life threatening (CTCAE v5.0)						

Supplemental Table 4. Inclusion and exclusion criteria, related to figures 1 and 2

**Inclusion criteria**
Men and Women and members of all ethnic groups who are at least 18 years old at the time of enrollment are eligible for this trial;
Informed consent obtained and signed;
Willing and able to commit to study procedures including long-term follow-up visit(s) on or off the study protocol;
Histopathologically confirmed 2016-WHO grade III recurrent glioma, and grade IV recurrent glioblastoma (GBM), inclusive of Gliosarcoma;
In all cases, the diagnosis must be confirmed by a pathologist.
Recurrent surgically resectable tumor and or biopsy;
Participants who have undergone surgical resection should have received an MRI or a scan after surgery in order to visualize residual tumor. If not, the operative report must be available;
Prior to surgery there was imaging evidence of measurable progressive disease (PD);
Re-radiation, if indicated, should occur at least 2 weeks after surgery and/or biopsy, once the wound has healed well without any drainage or cellulitis;
Estimated survival of at least 3 months;
Hgb > 9 gm; absolute neutrophil count (ANC) > 1500/μl; platelets > 100,000; creatinine < 1.5 times the upper limit of laboratory normal value; bilirubin < 2 times the upper limit of laboratory normal value; SGPT or SGOT < 3 times the upper limit of laboratory normal value;
Chemotherapy must start within 8 weeks of tumor resection or biopsy;
Bevacizumab (Avastin) is allowed. If indicated, it should be initiated at least 4 weeks post craniotomy or biopsy if the wound has healed well without any drainage or cellulitis;
The use of herbal preparation or tetrahydrocannabinol/cannabidiol is strongly discouraged, but not contraindicated;
**Exclusion criteria**
Subjects with newly diagnosed GBM
Pregnant women or nursing mothers. Women of childbearing age must have a negative pregnancy test prior to study entry. Women of childbearing potential must practice medically approved contraceptive precautions;
Abnormal hematological results at inclusion with neutrophils < 1,500/mm3 and/or blood-platelets < 100,000/mm3
Severe or chronic renal insufficiency (creatinine clearance ≤ 30 ml/min
Unable to adhere to required procedures, visits, examinations described in the study;
Any usual formal indication against imaging examinations (important claustrophobia, pacemaker);
History of another malignancy in the previous 2 years, with a disease-free interval <2 years. Patients with prior history of in situ cancer or basal or squamous cell skin cancer, any time prior to screening, are eligible.
OPTUNE device is not permitted in the study;
Participation in clinical trials utilizing a liquid biomarker or imaging studies that impact overall survival.
Abbreviations: ANC, absolute neutrophil count; GBM, glioblastoma; PD, progressive disease; SGOT, serum glutamate oxaloacetate transaminase; SGPT, serum glutamate pyruvate transaminase.

Supplemental Table 5. Examples of Limiting Dilution Tumorigenic Assays of patient-derived GBM of bioreactor-enriched CSCs, related to STAR methods

Immune-deficient mice were injected into the flank in the presence of matrigel with various doses of GBM bioreactor- enriched CSCs and tumor growth was followed for up to 14 weeks.

Primary cell line	N° of CSCs Inoculated	N° Tumors formed	Tumor Palpation weeks)
	$1x10^5$	5/5	5
BNC-1	$1x10^4$	5/5	8
	$1x10^3$	5/5	8-10
	$1x10^2$	4/5	8-12
	$1x10^1$	0/5	12-14
BNC-2	$1x10^5$	5/5	5
	$1x10^4$	5/5	8
	$1x10^3$	4/5	8-10
	$1x10^2$	3/5	8-12
	$1x10^1$	0/5	12-14
BNC-3	$1x10^5$	5/5	5
	$1x10^4$	4/5	8
	$1x10^3$	4/5	8-10
	$1x10^2$	4/5	8-12
	$1x10^1$	0/5	12-14
BNC-4	$1x10^5$	5/5	5
	$1x10^4$	5/5	8
	$1x10^3$	5/5	8-10
	$1x10^2$	4/5	8-12
	$1x10^1$	0/5	12-14
BNC-5	$1x10^5$	5/5	5
	$1x10^4$	5/5	8
	$1x10^3$	5/5	8-10
	$1x10^2$	3/5	8-12
	$1x10^1$	0/5	12-14
